# Group schema therapy for eating disorders: study protocol

**DOI:** 10.1186/s40337-017-0185-8

**Published:** 2018-01-09

**Authors:** Fiona Calvert, Evelyn Smith, Rob Brockman, Susan Simpson

**Affiliations:** 1 School of Psychology, University of Wollongong, Wollongong, NSW Australia; 2 School of Social Sciences and Psychology, Western Sydney University, 1795 Locked bag, Penrith, NSW Australia; 3 University of Technology Sydney, Ultimo, NSW Australia; 4 School of Psychology, Social Work & Social Policy, University of South Australia, Adelaide, SA Australia

## Abstract

**Background:**

The treatment of eating disorders is a difficult endeavor, with only a relatively small proportion of clients responding to and completing standard cognitive behavioural therapy (CBT). Given the prevalence of co-morbidity and complex personality traits in this population, Schema Therapy has been identified as a potentially viable treatment option. A case series of Group Schema Therapy for Eating Disorders (ST-E-g) yielded positive findings and the study protocol outlined in this article aims to extend upon these preliminary findings to evaluate group Schema Therapy for eating disorders in a larger sample (*n* = 40).

**Methods/design:**

Participants undergo a two-hour assessment where they complete a number of standard questionnaires and their diagnostic status is ascertained using the Eating Disorder Examination. Participants then commence treatment, which consists of 25 weekly group sessions lasting for 1.5 h and four individual sessions. Each group consists of five to eight participants and is facilitated by two therapists, at least one of who is a registered psychologist trained on schema therapy. The primary outcome in this study is eating disorder symptom severity. Secondary outcomes include: cognitive schemas, self-objectification, general quality of life, self-compassion, schema mode presentations, and Personality Disorder features. Participants complete psychological measures and questionnaires at pre, post, six-month and 1-year follow-up.

**Discussion:**

This study will expand upon preliminary research into the efficacy of group Schema Therapy for individuals with eating disorders. If group Schema Therapy is shown to reduce eating disorder symptoms, it will hold considerable promise as an intervention option for a group of disorders that is typically difficult to treat.

**Trial registration:**

ACTRN12615001323516. Registered: 2/12/2015 (retrospectively registered, still recruiting).

## Background

The treatment of eating disorders is a difficult endeavor, with only a relatively small proportion of clients responding to standard cognitive behavioural therapy (CBT). Less than half of those with bulimia nervosa (BN) have recovered at follow-up after receiving CBT [17, 18, 23] and research supporting cognitive-behavioural treatment for anorexia nervosa (AN) is limited, with no clear indication of improvement in this population [6, 8]. Approximately 50% of patients with eating disorders continue to be highly symptomatic at 60-week follow-up following transdiagnostic CBT [16]. Further, treatment dropout rates are high amongst individuals with eating disorders [9, 43] with one literature review reporting an average drop-out rate of between 20 and 51% in inpatient settings and between 29 and 73% in outpatient settings [20].

The treatment of eating disorders is especially complicated by a high level of co-morbidity [3]. Approximately 69% of individuals with eating disorders may meet DSM IV (APA, 1994) diagnostic criteria for a personality disorder and 93% of these clients may also have other co-morbidity including anxiety and substance use disorders. Eating disorders are also associated with the presence of rigid personality features, which increases clinical complexity and is associated with poorer treatment outcomes [22, 26, 46]. Eating disorders have also been linked to a range of trauma-related risk factors, including childhood abuse and neglect, which may also be mediated by personality disorder diagnoses [5]. Individuals with eating disorders also commonly experience complex and difficult-to-treat symptomatology including dissociation, perfectionism, compulsive pathology, rigid thinking patterns [28, 30, 38, 49] and high levels of shame [7].

Given the prevalence of co-morbidity and complex personality traits in this population, it is important to consider the deeper belief systems underlying eating disorder presentations. Schema Therapy ([53]/1999) is becoming an increasingly popular psychological model for working with individuals with complex mental health and personality difficulties. Schema Therapy combines aspects of cognitive, behavioral, experiential, interpersonal and psychoanalytic therapies into one integrative and unified model [1]. The schemas that are targeted in treatment are enduring and self-defeating patterns that typically begin early in life. These patterns consist of negative/dysfunctional thoughts and feelings which have been repeated and elaborated upon, and pose obstacles for accomplishing one’s goals and getting one’s needs met [40]. These schemas are perpetuated behaviorally through the coping styles of schema maintenance, schema avoidance, and schema compensation. The Schema therapy model of treatment is designed to help the person break these negative patterns of thinking, feeling and behaving and develop healthier alternatives to replace them [1].

The evidence for schema therapy for individuals with complex mental health difficulties is growing. This approach has been applied, in both individual and group forms, to a wide variety of clinical disorders, including, borderline personality disorder [19, 21] and chronic depression [11, 34, 41, 42]. A recent stringent systematic review found medium to large effect sizes for schema therapy in the treatment of a range of psychological conditions [35]. Attention has recently been given to the applicability of Schema Therapy to individuals with eating disorders (Pugh, 2015). Evidence suggests that maladaptive schemas are more strongly held by individuals with anorexia and bulimia nervosa compared to normal controls [30]. Preliminary data [33, 38] supports the notion that it is the schema processes that are engaged in an attempt to avoid intolerable emotional states associated with these schemas that in fact determine whether an individual will manifest restrictive or bulimic eating pathology. Whereas restrictive eating pathology may be a compulsive behavior developed to prevent schemas being triggered at all (schema compensation), bulimic pathology may function alongside other impulsive behaviors as a method of escaping schema-related affect once schemas have already been triggered (schema avoidance) [33, 38].

Schema therapy has been used in individuals with eating disorders in one preliminary study [44]. Simpson et al. examined the use of Group Schema Therapy for Eating Disorders (STE-g) in a case series of eight participants with chronic eating disorders and high levels of co-morbidity. Treatment was comprised of 20 sessions which included cognitive, experiential, and interpersonal strategies, with an emphasis on behavioral change. Clinically significant change was observed from pre-treatment to six-month follow-up for eating disorder severity (*d* = 1.70), global schema severity (*d* = 1.59), shame (*d* = 0.91), and anxiety (*d* = 1.53). Clinically significant change in eating disorder severity at follow-up was also shown for the majority of completers (six participants out of eight completed the full treatment program). Self-report feedback suggested that group factors may catalyze the change process in schema therapy by increasing perceptions of support and encouragement to take risks and try out new behaviors, whilst providing a de-stigmatizing and de-shaming therapeutic experience [44].

The present study aims to extend upon the preliminary findings of Simpson et al. [44] to evaluate Schema Therapy in a large eating disordered sample (*n* = 40), in terms of reduction of symptoms, and assess feasibility, acceptability and predictors of outcomes. We aim to conduct 6 groups of schema therapy in two locations: 1) University of South Australia in Adelaide Australia and 2) Western Sydney University in Sydney Australia. The study will examine whether Schema Therapy reduces eating disorder symptoms and improves psychological wellbeing and quality of life, both at post treatment and follow-up.

## Method

### Participants

Approximately 40 participants will be recruited into the study. All participants will be females aged 18 years and over, meeting *Diagnostic and Statistical Manual of Mental Disorders, 5th Edition* criteria for an eating disorder, following a transdiagnostic approach (Fairburn, et al., 2003). Participants are recruited via word of mouth, letters to clinicians and advertisements through *Facebook* as well as support organisations such as *The Butterfly Foundation.* Participants are provided with information about the study and, if they agree to participate, are required to give written consent. Full disclosure of the purpose of the study, the potential benefits and risks associated with participation, and the confidential nature of information obtained in the study is explained to participants.

### Inclusion/ exclusion criteria

Participants with active psychotic symptoms, high suicide risk or current crisis status (self-disclosed at baseline), a BMI of less than 14, intellectual disability, and those who are consuming large amounts of alcohol/drugs are excluded from the study. All participants must have a general practitioner involved in their care to monitor their physical health in order to participate in this research.

### Overall study design

This study will be an uncontrolled single group repeated measures design. Ethics approval was secured and participants provided informed consent to participate. Participants are first screened over the phone to ensure suitability for the group. If they agree to participate in the group, they then undergo a two-hour assessment where they complete a number of standard questionnaires and their diagnostic status is ascertained. Participants then commence treatment which consists of 25 weekly group sessions lasting for 1.5 h. Participants are also provided with four individual sessions that they can book with one of the therapists whenever they want. Each group consists of six to eight participants and is facilitated by two therapists, at least one of whom is a registered psychologist with training on schema therapy, and supervised by a schema therapist. Participants complete psychological measures and questionnaires at pre, post, 6 month and 1 year follow-up.

### Measures

This study utilises a battery of assessments conducted at baseline, mid-treatment, end-of-treatment, and six- and twelve-month follow-up points. These assessments are conducted by fully registered psychologists who have received specialized training in the administration of standardized eating disorder measures. Table 1. Provides a summary of these assessments.

**Table 1:** Assessments conducted at different time points

Measure	Baseline	Weekly	Mid	Post	6 months	12 months
EDE	x			x		
EDE-Q	x	x (abbrev)	x	x	x	x
YSQ-SF	x		x	x	x	x
SMI	x		x	x	x	x
WHO-5	x		x	x	x	x
CORE-10	x		x	x	x	x
SCS-SF	x		x	x	x	x
SATAQ	x		x	x	x	x
MCMI-III	x			x		
BSL-23	x			x	x	x

*Notes: EDE = Eating Disorder Examination; EDE-Q = Eating Disorder Examination- Questionnaire; YSQ-SF = Young Schema Questionnaire, short form; SMI = Schema Mode Inventory; WHO-5 = World Health Organisation-Five Well-Being Index; CORE-10 = Clinical Outcomes in Routine Evaluation-Outcome Measure; SCS = Self-Compassion Scale*- *Short Form; SATAQ = Sociocultural Attitudes Towards Appearance Questionnaire; MCMI-III = Millon Clinical Multiaxial Inventory- III; BSL-23 = Borderline Symptoms List*

#### *Eating Disorder Examination* (EDE; [15])

The EDE is a structured, investigator-based interview that measures the severity of symptoms of eating disorders. The scale can be used to ascertain an individual’s eating disorder diagnosis, as is the purpose of the measure in the current study. The EDE has been shown to have excellent reliability when administered by trained examiners [12].

#### *Eating Disorder Examination- Self-Report Questionnaire Version* (EDE-Q; [14])

The EDE-Q is a 36-item self-report questionnaire for the assessment and diagnosis of eating disorders. The EDE-Q yields four subscale scores—Restraint, Eating Concern, Weight Concern, and Shape Concern—as well as a global score, which is an average of all four subscales. The EDE-Q has been shown to have good convergent validity [10, 14]. Acceptable internal consistency and test–retest reliability have also been demonstrated [32, 37].

#### *Young Schema Inventory- Short Form* (YSQ-SF; [52])

The YSQ-SF is a self-report measure used to assess 15 different maladaptive schemas (emotional deprivation, abandonment, mistrust/abuse, social alienation, defectiveness, incompetence, dependency, vulnerability to harm, enmeshment, subjugation of needs, self-sacrifice, emotional inhibition, unrelenting standards, entitlement, and insufficient self-control). The scale consists of 75 items rated from one (*completely untrue of me*) to six (*describes me perfectly*). The scale has been shown to have good psychometric properties [25, 50].

#### *Schema Mode Inventory-Short Form* (SMI; [31])

The SMI measures the presence of 14 schemas modes: Vulnerable Child, Angry Child, Enraged Child, Impulsive Child, Undisciplined Child, Happy Child, Compliant Surrender, Detached Protector, Detached Self-Soother, Self-Aggrandizer, Bully and Attack, Punitive Parent, Demanding Parent and Healthy Adult modes. The questionnaire consists of 118 items which are given frequency ratings using a Likert scale ranging from one (never or hardly ever) to six (always). An overall score is calculated from the scale sum score divided by the number of items in that scale. The short form of the SMI has been shown to have acceptable internal consistencies amongst the 14 subscales (Cronbach α’s from .79 to .96) as well as adequate test-retest reliability and moderate construct validity [31].

#### *World Health Organisation-Five Well-Being Index* (WHO-5; [51])

The WHO-5 is an assessment of general wellbeing consisting of five statements (e.g. *I have felt cheerful and in good spirits* and *I have felt calm and relaxed*), which participants rate on a six-point scale (from *never* to *always*), with a possible total score varying from 0 to 25. Higher scores on the WHO-5 reflect better well-being.

#### *Clinical Outcomes in Routine Evaluation-Outcome Measure* (CORE-10; [2])

The CORE-10 is a self-report measure of general psychological distress. The CORE-10 includes 10-items which the respondent rates on a five-point Likert scale (from *not at all* to *most or all of the time*), for example *I have felt tense, anxious or nervous* and *I have felt panic or terror*. The CORE-10 has been shown to have good internal reliability (α = .90) and a correlation of.94 with the CORE-OM [2].

#### *Self-Compassion Scale*- *Short Form* (SCS-SF; [39])

The SCS-SF is a 12-item, self-report scale which assesses the positive and negative aspects of the three main components of self-compassion: Self-Kindness (e.g., *When I’m going through a very hard time, I give myself the caring and tenderness I need*) versus Self-Judgment (e.g., *I’m disapproving and judgmental about my own flaws and inadequacies*); Common Humanity (e.g., *When I feel inadequate in some way, I try to remind myself that feelings of inadequacy are shared by most people*) versus Isolation (e.g., *When I fail at something that’s important to me, I tend to feel alone in my failure*); and Mindfulness (*When something upsets me I try to keep my emotions in balance*) versus Over-Identification (*When I’m feeling down I tend to obsess and fixate on everything that’s wrong).* Responses are given on a five-point scale ranging from one (*almost never*) to five (*almost always*). A total self-compassion score is calculated as a mean of all items and higher scores correspond to higher levels of self-compassion. The SCS-SF has good psychometric properties, with high internal consistency (α = .85; [45]) and a very high correlation with the long form of the SCS [39].

#### *Sociocultural Attitudes Towards Appearance Questionnaire- Internalization subscale* (SATAQ; [24])

The SATAQ is a 14-item inventory assesses women’s recognition and acceptance of societally prescribed standards of physical appearance, particularly the thin ideal. The 8-item Internalization subscale is used in the present study and measures the extent to which the individual personally accepts these standards. Statements such as *I tend to compare my body to people in magazines and on TV* are rated from one (*completely disagree*) to five (*completely agree*). The SATAQ converges satisfactorily with other measures of body image and eating disturbance [24, 47].

#### *Millon Clinical Multiaxial Inventory- III* [36]

The MCMI-III is a psychological assessment that provides information on longstanding personality patterns and clinical symptomatology. The tool consists of 175 items that are scored on a *True/False* basis that are scored to produce 28 clinical subscales. Reliability and validity studies on the MCMI indicate that is generally a psychometrically sound instrument. The scale demonstrates good internal consistency with alpha coefficients of above.80 for the majority of the scales (manual). Test-retest reliability has been shown to be moderate to high [13, 29].

#### *Borderline Symptoms List* (BSL-23; [4])

The BSL-23 is a questionnaire used to assess the degree of symptoms of BPD, such as poor self-esteem, dysphoric emotions, suicidal intention and impulsive behaviors. The scale consists of 23 items (for example: *I experienced stressful inner tension* and *I wanted to punish myself*) rated on a five-point Likert scale from 0 (*not at* all) to 4 (*very strong*). A total score is obtained by summing responses and higher scores represent more severe BPD symptomatology. The BSL-23 has good psychometric properties with high internal consistency (α = 0.94–0.97) and the ability to discriminate personality disorder patients from patients with other clinical symptomatology (mean effect size of 1.13; [4]).

### Primary outcomes

Eating disorder symptom severity is the primary outcome of this, as measured by the EDE and EDE-Q. The EDE will be used for pre to post, but due to limited resources only the EDE-Q will be completed at follow-up.

### Secondary outcomes

The secondary outcomes measured in this study include: cognitive schemas (measured using the YSQ); self-objectification (measured using the SATAQ-Internalisation); general quality of life (WHO-5 and CORE-10); self-compassion (SCS-SF); schema mode presentations (measured using the SMI); and Personality Disorder features (measured using the MCMI-III and the BSL-23).

### Intervention

The schema therapy eating disorder group (STE-g; [44]) was based on the schema mode model, with some components drawing on the schema-therapy treatment program: “Schema Focused Therapy in a Group Setting” [48]. The program consists of twenty-five 90-min sessions. All participants are provided with a patient-version workbook which corresponds with the treatment manual. The first part of the group focuses on schema psychoeducation and schema-focused cognitive behavioral strategies which help participants to identify and start challenging their schemas, whilst working on behavioral change both within and outside the group. This model assists participants to develop an individualised formulation of their own difficulties using a schema therapy framework. The highest schemas and modes are identified for each patient on the basis of their scores on relevant measures. Each session is recorded and group members are strongly encouraged to come in to watch the video should they miss a session. This is to ensure that participants do not miss any educational material, and retain a sense of connectedness with the group.

Particular emphasis is placed on linking eating behaviors to the schema modes, so that participants are able to learn about the origins and coping functions associated with their own pattern of symptoms. Avoidant coping in the group is addressed frequently by labeling the “Detached Protector” mode when it is identified either by group leaders or by other participants (i.e., both in the context of the group and when talking about eating behavior which took place between sessions). Use of over-compensatory behaviors to avoid emotions is also identified and group members are encouraged to point this out to each other empathically as the group progresses. Due to the lack of emotional awareness and emotional tolerance within the group, participants are encouraged to learn to express empathy and to ask for their emotional needs to be met within the context of the group, rather than detaching from emotional needs through eating behaviors. Schema-focused mindfulness meditation [27], is incorporated to increase awareness of urges to carry out eating disordered behaviors, modes and associated triggers and to facilitate emotional regulation.

Participants are helped to identify the origins of their negative body-image, and to link these to the development of particular modes. Body-image restructuring work involves participants learning to recognise the way in which their perception and visceral sense of their bodies differs from the perspective of their most prominent modes, and developing a compassionate “Healthy Adult” mode which is accepting and nurturing toward the body. Mode dialogues are used to enable group members to repeatedly coach each other to send the “Critical” mode away when it expresses negative assertions about the body or self (e.g. “You are ugly, fat, and nobody really likes you”). This gives group participants the opportunity to practice being in Healthy Adult mode firstly for others and then for themselves. Flashcards are used to reinforce what is learned in sessions and to help participants to challenge modes and resist eating disordered behavior between sessions. In the four individual sessions the therapist ensure that the participant understand the schema model, manages crisis, and engages in schema therapy.

### Data analysis

Within-patient standardized effect size information (i.e., Cohen’s d) from baseline to end-of-treatment, and from baseline to follow-up will be calculated for eating psychopathology, and all secondary outcomes. Clinical outcome data will be analysed using intention to treat criteria. Data from outcome measures will be analysed with paired sample T-test. To assess group differences between the two treatment sites, independent t-tests will be used. The effect size of outcome measures will be compared to the effect size reported for previous studies CBT for eating disorders. The criterion used for statistical significance will be *p* < 0.05. Sample size was estimated assuming 80% power, significance level at 0.05, and an effect size of 0.5; 26 people are needed. Assuming a 50% drop out as documented in previous CBT trials, we will need around 40 people.

At the completion of the treatment, a random set of participants will be invited to provide qualitative feedback on their experiences of the group treatment. These interviews will be one-to-one, and conducted either face-to-face, or via telephone, and are expected to last for between 30 and 60 min. Transcripts of these interviews will be made and the data will be analysed using a thematic analyses approach. This information will be used to provide preliminary information about treatment efficacy and acceptability, and inform the design of future studies.

### Discussion

This study will expand upon preliminary research of group Schema Therapy for individuals with eating disorders [44]. If group Schema Therapy is shown to reduce symptoms for this population, it will hold considerable promise as an intervention option for a group of disorders that is typically difficult to treat.

As this is an uncontrolled study it is limited by not having a proper comparison to control for confounding variables. We initially designed this study with a control group, group Cognitive Behaviour Therapy (CBT), but then were unable to recruit participants for the CBT group in Adelaide, and participants only wanted to take part of the research if they were in the schema therapy group. This was mostly due to the fact that Adelaide is a small city and referrals were usually received for severe cases, as most of the participants so far had completed CBT before. We do wish to conduct a randomized controlled trial comparing schema therapy to CBT, but that will need to be conducted in Sydney.

Since the groups are transdiagnostic this has a few advantages and disadvantages. First, the transdiagnostic approach for eating disorders is well established, it helps having different eating disorders as this will provide variety in the group on schemas and modes, which might facilitate engagement. However, within the group people might feel very dissimilar to each other within a transdiagnostic approach, and the psychologists running the groups will keep this in mind to bring unity to the group by articulating commonalities among participants.

This study will be the first to modify schema therapy to fit the eating disorders sample and to deliver it to this group. This uncontrolled study will establish proof of concept and assess feasibility, acceptability and potential harms. To date, the study has completed four 25-week group treatments and has commenced a further two groups.
